# Internal Regulation Center in hospitals: Repercussions of its implementation on the health services’ indicators

**DOI:** 10.1590/1518-8345.5700.3517

**Published:** 2022-03-21

**Authors:** Vivian Biazon El Reda Feijó, Maynara Fernanda Carvalho Barreto, Marcos Tanita, Alexandre Pazetto Balsanelli, Isabel Cristina Kowal Olm Cunha, Maria do Carmo Fernandez Lourenço Haddad

**Affiliations:** 1 Universidade Estadual de Londrina, Hospital Universitário de Londrina, Londrina, PR, Brasil.; 2 Universidade Estadual do Norte do Paraná, Setor de Enfermagem, Bandeirantes, PR, Brasil.; 3 Universidade Federal de São Paulo, Departamento de Administração em Serviços de Saúde e Enfermagem, São Paulo, SP, Brasil.; 4 Bolsista do Conselho Nacional de Desenvolvimento Científico e Tecnológico (CNPq), Brasil.; 5 Universidade Federal de São Paulo, Departamento de Enfermagem, São Paulo, SP, Brasil.; 6 Universidade Federal de São Paulo, Departamento de Enfermagem, São Paulo, SP, Brasil.

**Keywords:** Health Evaluation, Benchmarking, Quality Management, Hospitalization, Health Status Indicators, Hospital Bed Capacity, Avaliação em Saúde, Benchmarking, Gestão da Qualidade, Hospitalização, Indicadores Básicos de Saúde, Número de Leitos em Hospital, Evaluación en Salud, Benchmarking, Gestión de la Calidad, Hospitalización, Indicadores de Salud, Capacidad de Camas en Hospitales

## Abstract

**Objective:**

To evaluate the hospital indicators and their repercussions on the number of monthly admissions to a public university hospital, before and after implementing the Internal Regulation Center.

**Method:**

An evaluative research study, of the Case Study type, developed in a public university hospital. A total of 28 indicators related to structure, production, productivity and quality were measured, which are part of internal Benchmarking. The data were analyzed by means of descriptive statistics and multiple regression to identify the independent factors and those associated with the number of monthly hospitalizations with 95% confidence intervals.

**Results:**

Implementation of the Center significantly increased (p<0.001) the number of discharges, the bed utilization factor and the bed renewal rate, emergency hospitalization, bed occupancy percentage, surgical procedures performed and the patient-day mean value (p=0.027). There was a reduction (p<0.001) in the number of visits to the medical, obstetric and orthopedic emergency room, in the rates of in-hospital infection and infant mortality, as well as a mean reduction of 0.81/day, approximately one day less of hospitalization per patient, or a gain of 40 available beds per month.

**Conclusion:**

Although the number of available beds was lower in the post-implementation period, the bed replacement interval was reduced, representing an increase of 40 more beds per month due to the reduction in the patients’ length of stay in the institution.

## Resumo

**Objetivo:**

Avaliar os indicadores hospitalares e suas repercussões, antes e após a implantação do Núcleo Interno de Regulação, no número de internações mensais em hospital universitário público.

**Método:**

Pesquisa avaliativa, do tipo Estudo de Caso desenvolvida em hospital universitário público. Foram mensurados 28 indicadores relacionados à estrutura, produção, produtividade e qualidade, que integram o referencial de *Benchmarking* interno. Os dados foram analisados por estatística descritiva e regressão múltipla para identificar os fatores independentes e associados ao número de internações mensais com intervalos de confiança de 95%.

**Resultados:**

A implantação do Núcleo aumentou significativamente (p<0,001) o número de altas, o fator de utilização e índice de renovação dos leitos, internação de urgência, porcentagem de ocupação dos leitos, procedimentos cirúrgicos realizados e média de paciente-dia (p=0,027). Houve redução (p<0,001) no número de atendimentos no pronto socorro médico, obstétrico e ortopédico, nas taxas de infecção hospitalar e de mortalidade infantil, bem como na diminuição média de permanência de 0,81/dia, aproximadamente um dia a menos de internação por paciente, ou um ganho de 40 leitos disponíveis ao mês.

**Conclusão:**

Embora o número de leitos disponíveis tenha sido menor no período pós-implantação, o intervalo de substituição de leitos reduziu, representando o aumento de mais 40 leitos ao mês devido à diminuição do tempo de permanência dos pacientes na instituição.

## Resumen

**Objetivo:**

Evaluar los indicadores hospitalarios y sus repercusiones, antes y después de la implantación del Centro Interno de Regulación, sobre el número de internaciones mensuales en un hospital universitario público.

**Método:**

Investigación evaluativa, del tipo Estudio de Caso, desarrollada en un hospital universitario público. Se midieron 28 indicadores relacionados con la estructura, producción, productividad y calidad, que forman parte del *Benchmarking* interno. Los datos fueron analizados por estadística descriptiva y regresión múltiple para identificar factores independientes y asociados con el número de hospitalizaciones mensuales con un intervalo de confianza del 95%.

**Resultados:**

La implantación del Centro incrementó significativamente (p<0,001) el número de altas, el factor de utilización y tasa de renovación de camas, la hospitalización de urgencia, el porcentaje de ocupación de camas, los procedimientos quirúrgicos realizados y el promedio de pacientes/día (p =0,027). Se registró una reducción (p<0,001) en el número de las consultas de emergencias médicas, obstétricas y ortopédicas, en las tasas de infección hospitalaria y mortalidad infantil, además de una disminución promedio de la estancia del 0,81/día, aproximadamente un día menos de hospitalización por paciente, o 40 camas disponibles más mes.

**Conclusión:**

Aunque el número de camas disponibles fue menor en el período posterior a la implantación, el intervalo de sustitución de camas se redujo, lo que representó un aumento de 40 camas más por mes debido a la disminución de la estancia de los pacientes en la institución.

**Highlights:**

(1) Implementation of the IRC significantly increased the number of hospital discharges.

(2) There was a reduction in the in-hospital infection rate after implementing the IRC.

(3) Implementation of the IRC resulted in one day less of hospitalization per patient.

(4) Implementation of the IRC resulted in a gain of 40 beds available *per* month.

(5) The bed replacement interval was reduced after implementing the IRC.

## Introduction

The demand for care and access to health has increased and is faced with a scenario in which the available resources, such as beds, are scarce. In view of this reality, rationalizing the hospital structure becomes a priority objective of public and private hospital institutions in order to guarantee their sustainability^1^.

The need to optimize the use of hospital beds occurs as a result of their scarcity to meet the health demands and, therefore, it becomes necessary to implement strategies to improve the performance of hospital services, with the bed occupancy rate indicator being indicated to measure efficiency and productivity in hospitals, especially public ones^2^ ^-^ ^4^.

According to the National Health Survey carried out by the Brazilian Institute of Geography and Statistics (*Instituto Brasileiro de Geografia e Estatística*, IBGE) in 2019, among the people who required hospitalization for 24 hours or more, 64.6% (8.9 million) were treated by institutions belonging to the Unified Health System (*Sistema Único de Saúde*, SUS)^5^.

Bed management through the monitoring of indicators, associated with other information and governance measures, provides indispensable data to support discussions on the profile and flow of patients treated^6^, on the rational use of hospital resources and on the promotion of comprehensive health care^7^.

A study including 25 public hospitals in Iran revealed the importance of health managers acting in the implementation of strategic actions with a view to improving access and universal health coverage and, consequently, increasing the bed occupancy and bed turnover rates, as well as reducing the mean length of stay and the in-hospital mortality rate^8^.

Regarding the mean length of stay indicator, as an indicator of institutional efficiency, it is also used to dimension the bed infrastructure necessary to meet a given demand, and the shorter the length of stay, the lower the number of beds, as a greater number of patients can be hospitalized in each bed^9^.

In this context, with the objective of establishing guidelines for the organization of the hospital component of the Health Care Network (*Rede de Atenção à Saúde*, RAS), the Brazilian Ministry of Health (*Ministério da Saúde*, MS) published the National Hospital Care Policy (*Política Nacional de Atenção Hospitalar*, PNHOSP), within the scope of the SUS, and the creation of Internal Regulation Center (IRC) in hospitals made it possible to organize access to consultations and to diagnostic and therapeutic services, as well as to beds available for hospitalization^10^.

The IRC concerns an institutional coordination instance, which has the purpose of centrally managing beds, in addition to acting as an interface between the Health Units and the Regulation Centers of access to health care, integrated and agreed upon^10^. In public hospitals, the role of the IRC in the operationalization, organization and monitoring of hospital process and outcome indicators is extremely relevant, as it strengthens the objectives of the SUS Institutional Support and Development Program (*Programa de Apoio e Desenvolvimento Institucional do SUS*, PROADI-SUS)^11^.

Implementation of the IRC, in a medium and long term, results in the improvement of hospital indicators, as well as in a significant reduction in the number of patients admitted directly to the surgical center and referred to the urgency and emergency sector in the postoperative period, in addition to reducing the occurrence of patients returning to the urgency and emergency sector in the postoperative period^12^.

Considering that performance of the IRC promotes an improvement in institutional efficiency, which can be measured by monitoring the institutional performance indicators, it is correct to assert that the same indicators can be used to assess IRC effectiveness, with regard to an efficient use of inpatient beds and in regulating access to the clinical and surgical ward beds^1^. Therefore, verification of the effectiveness of the actions implemented by the IRC and its repercussions in the practices and in the care and management processes is important, especially in public university hospitals, due to the magnitude of the care services provided by these institutions and their representativeness in the implementation of the SUS Health Public Policies.

In this perspective, Benchmarking, or comparative evaluation, constitutes a recommended tool to measure health indicators, which can be adopted as a framework to internally evaluate products and work processes (internal Benchmarking) or to compare them with other services (functional Benchmarking)^13^.

With regard to internal Benchmarking, it can be operationalized through structure indicators (planned, operational, idle capacity and number of operating rooms), production indicators (number of visits, number of hospitalizations), productivity indicators (replacement interval index, mean hospital stay) and quality indicators (rate of complications, hospital infection rate), in addition to other indicators such as economic-financial and image indicators^13^.

Thus, when implementing strategies that propose changes in the management of the care work, it is recommended to use indicators to measure the results after the intervention proposed^14^ and, considering that there is no standardization for bed management in Brazilian public hospitals^7^, this study aims at evaluating the hospital indicators and their repercussions, before and after the implementation of the Internal Regulation Center, on the number of monthly admissions to a public university hospital.

## Method

### Study design

This is an evaluative survey^15^, of the Case Study type.

### Data collection locus

Data collection took place in a public teaching hospital from the city of Londrina, PR, Brazil, a medium- and high-complexity care reference for 96 municipalities and an estimated population of 1,695,012 individuals. It is a tertiary-level hospital, with an installed capacity of 284 beds, of which 41 are intensive care beds and 243 are clinical backup beds, available in their entirety for SUS care.

### Period

The study was developed between January 2019 and July 2020.

### Selection criteria

The study was carried out from the implementation of the IRC in a public university hospital in southern Brazil. Implementation of the IRC took place in July 2016, based on the principles and guidelines set forth in the National Hospital Care Policy (PNHOSP)^10^.

The data referring to the hospital indicators were included in the study, in addition to those from the management of the hospital under study, referring to the period of 30 months before (from January 2014 to June 2016) and 30 months after (from July 2016 to December 2018) implementing the IRC. Definition of this period was based on the global data availability in the management information system, which took place starting from January 2014.

### Study variables

The variables collected integrate the internal Benchmarking framework^13^, adopted to internally evaluate products and processes, categorized into indicators of structure, production, productivity and quality, as follows: *structure* - non-extra beds available; *production* - hospital discharges, emergency room visits (surgical, burns, medical, obstetric, orthopedic and pediatric), admissions by internal transfer, total hospitalizations, patient-day mean value, inpatient surgical patients, elective surgical patients and occupancy percentage; *productivity* - bed utilization factor, renewal or turnover rate, emergency and elective hospitalizations, replacement interval, mean hospital stay and surgery suspension rate; and *quality* - maternal mortality ratio *per* 100,000, in-hospital infection rates, and general, infant, institutional and postoperative mortality rates.

It is noteworthy that these indicators and their respective classes are recognized by the Ministry of Health for the measurement and monitoring of epidemiological, quality and hospital management processes and results, subjected to data comparison at the national and international levels^11^.

### Data collection

Data collection took place through the monthly report made by the Medical Archive and Statistics Division of the hospital under study, in addition to recording the hospital variables and unprocessed numbers, referring to the period of 30 months before and 30 months after implementing the IRC.

### Data treatment and analysis

After collecting and preparing the database, the statistical analysis was performed using the MedCalc®19.5.1 statistical program, and the results of the continuous variables were described as mean (Me), standard deviation (SD) or median (Md) and interquartile range (IQR), depending on data distribution. The Student’s t test was used to compare the means of the continuous variables with normal distribution and homogeneity of variances, and the non-parametric test (Mann-Whitney’s U test) was applied to data with non-normal distribution and/or heterogeneity of variances. The statistical significance level used was 5%.

In order to complement the exploratory analysis and determine the association between calculated hospital management indicators, Pearson’s test was used. The variables with non-normal distribution were submitted to logarithmic transformation (Log). Multiple regression was performed to examine the relationship between the dependent variable (gross number of monthly hospitalizations) and the hospital management indicators. The indicators were selected considering the lowest probability of collinearity. For this, the Variance Inflation Factor (VIF) was used.

### Ethical aspects

Development of the study complied with the national and international research ethics standards, including approval by the Research Ethics Committee on April 24^th^, 2018, according to Opinion No. 2,618,220.

## Results

The comparative analysis of the IRC pre- and post- implementation phase showed a significant increase in the results of the production indicators: number of hospital discharges, bed utilization factor, bed occupancy percentage, bed renewal rate, hospitalizations from the emergency sector, number of admissions of elective surgical patients and number of surgical procedures performed.

Regarding the indicators that presented a decrease in their results, the number of visits to the medical, obstetric and orthopedic emergency room was lower in the IRC post-implementation period. The mean hospital stay, in-hospital infection and infant mortality rates also presented lower values, as shown in Table 1.

**Table 1:** Analysis of the IRC pre- and post-implementation hospital indicators with normal distribution. Londrina, PR, Brazil, 2019

Analysis variable	Pre-implementation		Post-implementation		p-value*
	Me	SD (±)	Me	SD (±)	
**Production Indicators**					
Hospital discharges	877.70	75.68	1,006.67	124.71	<0.001
Visits to the Burns Emergency Room	23.40	6.82	21.70	8.12	0.384
Visits to the Medical Emergency Room	475.23	69.46	367.87	49.75	<0.001
Visits to the Obstetric Emergency Room	839.60	109.38	674.40	51.05	<0.001
Visits to the Orthopedic Emergency Room	129.07	25.44	103.40	19.34	<0.001
Visits to the Surgical Emergency Room	523.00	67.8	483.97	83.08	0.051
Elective surgical patients	1.79	0.18	2.53	0.24	<0.001
Hospitalized surgical patients	508.17	45.22	672.43	73.44	<0.001
Occupancy percentage	83.87	4.70	92.41	5.88	<0.001
Surgical procedures in hospitalized patients	582.47	70.63	887.43	129.28	<0.001
**Productivity Indicators**					
Bed utilization factor	92.81	6.63	103.05	7.82	<0.001
Renewal index or Turnover rate	3.32	0.31	4.06	0.35	<0.001
Hospitalizations from the Emergency sector	785.00	69.5	925.87	104.21	<0.001
Elective hospitalizations	159.70	48.9	154.9	56.46	0.726
Mean hospital stay	7.72	0.57	6.91	0.46	<0.001
Surgery suspension rate	33.94	8.43	30.55	5.08	0.065
**Quality Indicators**					
In-hospital infection rate	10.15	1.61	8.01	1.13	<0.001
Overall mortality rate	6.88	0.93	6.99	0.87	0.628
Infant mortality rate	5.39	2.53	4.07	2.12	0.033
Institutional mortality rate	6.11	0.89	5.97	0.73	0.524
Postoperative mortality rate	2.16	0.53	2.24	0.68	0.646

^*^Student’s t test.

In relation to the variables with non-normal distribution, the number of visits to the pediatric emergency room, admissions by internal transfer, total hospitalizations, bed replacement interval, available beds and patient-day mean value presented differences between the periods of analysis. Admissions by internal transfer and total hospitalizations were higher in the IRC post-implementation period. It is noteworthy that, although the number of available beds was lower in the post-implementation period, the bed replacement interval was reduced (Table 2).

**Table 2:** Analysis of the IRC pre- and post-implementation hospital indicators with non-normal distribution. Londrina, PR, Brazil, 2019

Analysis variable	Md	IQR	Md	IQR	p-value*
**Structure Indicators**					
Beds available (non-extra)	284.00	276-294	261.00	258-271	0.001
**Production Indicators**					
Visits to the Pediatric Emergency Room	533.50	470-607	328.00	304-354	<0.001
Admissions by internal transfer	786.00	710-812	938.50	863-1079	<0.001
Total hospitalizations	966.50	908-992	1,069.50	1,017-1160	<0.001
Patient-day mean value	238.55	229.33-246.57	243.52	237.87-253.10	0.027
**Productivity Indicators**					
Replacement interval	1.45	1.13-1.79	0.46	0.29-0.80	<0.001
**Quality Indicators**					
Maternal mortality ratio per 100,000	0	0-0	0	0-0	0.615

*Mann-Whitney’s U test

As for the hospital indicators, there was a significant correlation (p<0.001) between the bed utilization factor and the renewal index or turnover rate, replacement interval (Log) and available beds (Log). The renewal index or turnover rate correlated (p<0.001) with the replacement interval for available beds (Log), available beds (Log) and mean hospital stay.

The bed replacement interval presented a correlation (p<0.001) with available beds (Log) and mean hospital stay. The occupancy percentage presented a correlation (p<0.001) with the following variables: bed utilization factor, renewal or turnover rate of available beds, bed replacement interval (Log), available beds (Log) and mean length of stay in the hospital (p=0.003). Table 3 shows the multiple regression performed to identify the indicators independently associated with the number of monthly hospitalizations.

**Table 3:** Multiple regression and hospital indicators independently associated with the number of monthly hospitalizations. Londrina, PR, Brazil, 2019

Hospital Indicator	Coefficient	Standard Error	t-student	p-value	R^partial^	R^semipartial^	VIF
Constant	1,039.9987						
Renewal index or Turnover rate	261.2798	9.663	27.038	<0.001	0.963	0.931	1.11
Beds available (non-extra)	3.9541	0.255	15.493	<0.001	0.899	0.533	1.11

^*^R^2^ determination coefficient: 0.9325; Adjusted R^2^: 0.9301.

Multiple regression showed that the renewal index or turnover rate, as well as the number of available non-extra beds were the indicators independently associated with the number of monthly hospitalizations (p<0.001), that is, it is possible to state that turnover of the existing beds was responsible for the increase in the number of hospitalizations, in the sense of guaranteeing access to a greater number of patients.

## Discussion

Implementation of the IRC in the hospital under study constituted an innovative management strategy, with relevant contributions to the health service, as the actions implemented exerted an impact on institutional performance, in the sense of greater efficiency, which can be proven from the results of the hospital indicators after its implementation.

From the results of the hospital indicators of the institution under study, an improvement in institutional performance is verified, which was possible from the development of work processes related to internal and external bed regulation, aimed at optimizing use of the existing beds.

The work processes developed by the IRC, based on the optimization of bed use, resulted in maintenance of the occupancy rates at satisfactory levels, with a reduction in the mean hospital stay. The result was a higher bed turnover rate and, consequently, greater availability of beds for the RAS, expanding this interface between internal and external regulation^11^ ^,^ ^16^. 

With the increase in the bed turnover rate and the reduction in the replacement interval, there is also an increase in the internal transfers of patients, interunits. Thus, an opportunity was verified to improve care processes related to patient safety in the transition of care and, this time, the hospital under study instituted SBAR as an ancillary tool to guarantee communication quality in the patient’s transition between the care units^17^. 

A study carried out with data from more than one million patients showed that, in addition to improving the performance of institutional indicators, adequate bed management promotes a reduction in the health services expenses^18^. 

In addition to that, the authors emphasize that there is a direct relationship between the best use of the available beds and the increase in the number of hospitalizations, which is in line with the results found in the current study^18^. Therefore, it is reasserted that, in a context of insufficient hospital beds, given the growing health demand of the population, bed management practices aimed at optimizing the installed capacity make it possible to guarantee access to health care for a greater number of patients.

Corroborating the above, a study carried out with the objective of describing the results achieved in the hospital performance indicators and in the bed supply, from the incorporation of a clinic management service, including internal bed regulation by the IRC, resulted in an annual increase in the number of hospitalizations, in the number of patients discharged home, an in the bed turnover rate, and in reductions in the mean length of stay and in in-hospital mortality^7^.

In relation to the “number of hospital discharges”, “bed utilization factor”, “bed occupancy percentage”, “number of surgical procedures performed”, “number of admissions by internal transfer” and “total number of hospitalizations” indicators, they also presented an increase in the IRC post-implementation period, proving that bed management actions are the basis for optimizing the use of the available resources^7^.

In the institution under study, bed management actions could be enhanced from their interrelation with the clinical management actions, among which the following can be mentioned: welcoming and risk classification of patients treated in the emergency units, existence of referral teams in the hospital units and use of tools to monitor the mean length of stay of hospitalized patients (Kanban), among others^7^ ^,^ ^11^.

Specifically with regard to length of stay, with the implementation of the IRC in the hospital under study, it was possible to improve the time spent by the patients in the urgency and emergency service, directly contributing to reducing overcrowding at the entrance door of the service. Monitoring the flow of patients in the urgency and emergency service is part of the IRC list of daily activities, and reducing the waiting time for an inpatient bed is a goal that aims at increasing access to the health service and efficiency in the use of hospital beds^19^ ^-^ ^20^.

As a result of maximizing institutional performance through the direct action of the IRC in the internal and external regulation of available beds with a view to maintaining the occupancy rate at adequate levels and reducing the mean stay from actions linked to the management of the clinic and a responsible discharge, it is also possible to positively interfere in the reduction of costs and expenses in the health services^4^, in addition to the qualification of the care provided and patient safety^21^ ^-^ ^22^.

With regard to the mean length of stay, studies show that a one-day reduction in the length of hospital stay, for a hospital with 300 beds, results in an expansion of the installed capacity of beds for effective use in a proportion of 49 new beds^16^. Considering that the results showed that the reduction in length of stay in the institution under study was 0.81/day, this represents an operational gain of 40 available beds each day.

Corroborating the assertion that the mean length of stay is an indicator that can be managed by the IRC, a study carried out in Thailand showed that prioritizing care, especially for aged individuals, optimizing the time between requesting and carrying out laboratory tests, and classifying the risk of the treated patients exerted a positive influence on the hospital stay indicator, reducing the number of hospitalization days and, therefore, it should be monitored to measure the performance of the emergency service^23^.

As for the “number of elective surgical patients” and “number of surgical patients admitted from the emergency sector” indicators, the bed management actions developed by the IRC in the planning and scheduling of surgeries are essential to guarantee access to the necessary care, minimizing the risk of delay and/or suspension of the scheduled surgical procedure^12^.

A study carried out in 2016, with data from the Hospital Foundation of the State of Minas Gerais in Brazil, analyzed 17,721 clinical and surgical consultations by the SUS in five general hospitals. Among these, 8,927 were admissions from the medical clinic and 8,794 from the surgical clinic, in which 40.6% of the hospitalized patients underwent surgical procedures. Knowing and managing this information is essential for the planning activities to enhance supply and access to the Brazilian^24^ public health system services^25^.

As for the reduced number of visits to the medical, obstetric and orthopedic emergency room in the post-implementation period, despite the strike periods between February and March 2015, May and June 2015 and October and November 2016, there was a reduction in the number of urgency and emergency visits, which could be explained by the improvement of the internal and external regulation practices, with direct repercussions on the dialog with the RAS, from insertion of the IRC in the management of transfer requests via the Hospital Bed Center and Urgency and Emergency Center, with a reduced response time to the regulatory services’ demands. However, confirmation of this hypothesis requires the development of complementary studies.

In the period comprised for the development of this study, in addition to periods of institutional strike, there was a 6.49% reduction in the number of available beds, after implementing the IRC, due to reforms and physical restructuring to qualify the care spaces and, even so, it was possible to significantly increase the number of monthly hospitalizations, from the enhancing of the existing infrastructure through the bed management actions carried out by the IRC. It is noteworthy that maintenance of the occupancy rates within established and safe limits to meet the health demands^1^ was a guideline leading decision-making in these periods.

In relation to the lower infection and infant mortality rates in the period after implementing the IRC, the thesis is defended that the actions and strategies implemented by the IRC, which resulted in a reduction of the time for patients to access the necessary care, with improvements in the care practices, may have contributed to the reduction of these two rates; however, it should be noted that these indicators are under a multifactorial influence and that the improvement of the management practices indirectly contributes to the improvement of institutional performance related to infection prevention and reduction of mortality.

In this sense, a study carried out with the objective of describing the process to develop and implement the reform of access to the emergency services and evaluating the effects on patient flow and indicators at the Princess Alexandra Hospital, in Australia, over 12 months, showed a reduction in mortality from 2.3% to 1.7%, less overcrowding and greater satisfaction of the users treated^26^.

It is observed that the shorter hospital stay can be related to lower infection rates and increased access to the health system by the users. Thus, reducing the mean length of stay indicates greater problem-solving capacity for the care and management team. It is noteworthy that the mortality indicator has the characteristic of measuring care quality and, when there is a reduction, it can represent an improvement in the care provided^18^.

Mortality rates in hospitals are directly related to delays in accessing the necessary care and to the unavailability of installed capacity to meet the patients’ needs. Thus, strategies that facilitate the flow of patients are necessary to reduce service queues and to increase the percentage of hospitalized patients according to their risk classification and waiting time^27^. In this study, the overall mortality rate remained unchanged in the period after implementing the IRC, despite the improvements achieved in the patient flows, proving that other factors are involved in obtaining these results.

Although the number of available beds was lower in the post-implementation period, the bed replacement interval was reduced, showing that the strategies used for bed management allow for equitable and transparent measures to meet the health users’ demands^7^.

Regarding the renewal or turnover rate, balancing the supply and demand of hospital services is one of the objectives of bed management^7^. In addition, bed management with the objective of using them efficiently is related to the control of hospital capacity, which allows for adequate bed turnover, in addition to ensuring patient safety^1^.

The results of this study showed that implementation of the IRC and the repercussion of bed management on the care and management work processes brought significant improvements in health care and, consequently, made existing beds available more efficiently, increasing the population’s access to health care necessary services.

It is noteworthy that the results identified in this research correspond to the wishes of the general population, managers, professionals, patients and students. The context of imbalance between demand and supply of inpatient beds generates the need for management strategies aimed at efficiency, but also at care quality and user’s satisfaction. Proof of this is that the waiting time for emergency care is one of the main indicators of satisfaction in users seeking care in this sector^28^.

The results presented can be precursors of a movement towards the optimization of bed management, so that elective patients enter hospitals at a moment as close as possible to the scheduled procedure, thus avoiding early hospitalization or urgency of care, as a strategy to guarantee the installed capacity insofar as it contributes to the qualification of hospital discharge, to the improvement of referral and counter-referral actions in the RAS, and to care continuity and completeness.

It is added that the strike periods in the institution under study and the temporary reduction in the number of beds available in the IRC post-implementation period represent limitations for the statistical analyses due to the difference between the periods evaluated. Although the type of analysis of the periods before and after implementation of the IRC does not allow controlling these factors, the results found showed that there was an improvement in institutional performance, which proves the importance of the actions that were implemented.

Non-assessment of the financial impact indicators is also considered as a limitation of this study. Therefore, new research studies are suggested that can assess the budgetary and financial impacts for the health institutions, from implementation of the IRC, as well as develop the stratification of the measured indicators in order to identify the potential for improvement by area and/or specialties such as adults’ health, maternal and children’s health, urgency and emergency, elective patients and outpatient care.

## Conclusion

Implementation of the IRC resulted in an increase in the number of monthly hospitalizations, despite a reduction in the number of available beds. The renewal rate and the number of available non-extra beds were hospital indicators independently associated with the number of monthly hospitalizations. It is noteworthy that an effective implementation of the IRC in the practice must be carried out by a multiprofessional team linked to the various areas related to the flows of hospital admission.

There was a daily operational gain of 40 beds resulting from the reduction in the mean length of stay by approximately one day. The results contribute to scientific advances in the area of care quality management, highlighting the importance of before-and-after research studies to assess the effectiveness of an intervention
